# Structural Insights into Arginine Kinase and Phosphagen Kinase Homologs: Mechanisms of Catalysis, Regulation, and Evolution

**DOI:** 10.3390/biology14091176

**Published:** 2025-09-02

**Authors:** Sung-Min Kang

**Affiliations:** College of Pharmacy, Duksung Women’s University, Seoul 01369, Republic of Korea; smkang@duksung.ac.kr

**Keywords:** arginine kinase, phosphagen kinase, protein structure, catalytic mechanism

## Abstract

Phosphagen kinases are vital for energy buffering and ATP regeneration in cells with high or fluctuating energy demands. Phosphagens are small, high-energy phosphate-storage molecules, such as arginine phosphate or creatine phosphate, that serve as immediate phosphate donors for rapid ATP production. Among them, arginine kinase plays a central role in invertebrates, while creatine kinase is predominant in vertebrates. This review presents a comprehensive structural analysis of arginine kinases and their homologs across diverse species, ranging from invertebrates like *Daphnia magna*, *Scylla paramamosain*, and *Limulus polyphemus* to the bacterial kinase McsB from *Staphylococcus aureus*. High-resolution crystal and cryo-EM structures reveal a common two-domain architecture and shed light on substrate-induced conformational changes, domain cooperativity, and catalytic mechanisms. Mutational studies highlight conserved residues such as His284 and their impact on enzyme dynamics. Importantly, the structure of bacterial arginine kinase-like kinases, such as McsB, unveils regulatory mechanisms mediated by activators like McsA. This structural diversity and functional specificity underscore the evolutionary adaptability of phosphagen kinases and their relevance as potential drug targets or diagnostic markers.

## Simple Summary

This study explores how a group of energy-related proteins called kinases work in a wide variety of animals and bacteria. These proteins help cells store and reuse energy quickly, especially during stress or activity. By comparing the detailed 3D structures of these proteins from different species, we identified common patterns and unique differences. These insights help explain how the proteins function and evolve. Understanding them can support new strategies in medicine, such as allergy control or developing treatments that target harmful organisms without affecting humans.

## 1. Introduction

Phosphagen kinases are a family of enzymes responsible for rapid ATP regeneration in cells that experience fluctuating or high energy demands [1]. These enzymes catalyze the reversible transfer of a phosphate group between ATP and a guanidino compound, known as a phosphagen. Among the most well-known members of this family are arginine kinase, which predominates in invertebrates, and creatine kinase, which is mainly found in vertebrates [2,3]. Despite differences in substrate preference and phylogenetic distribution, these kinases share a highly conserved structural fold and catalytic mechanism [4,5]. Understanding their structural variations across species offers valuable insights into energy metabolism, enzyme regulation, and potential biomedical applications.

Regulating the energy level is critical for the survival of all living cells. Members of the structurally and functionally related kinase family play an essential role in maintaining constant intracellular levels of Adenosine Triphosphate (ATP) [6]. Arginine kinase is a phosphotransferase that catalyzes the reversible transfer of a phosphate group between arginine and ATP/ADP, thereby interconverting arginine and phosphoarginine [7]. Phosphoarginine serves as a key energy-storage molecule, capable of rapidly donating its high-energy phosphate to ADP for ATP regeneration [8]. Phosphagens provide immediate energy during sudden increases in cellular activity, bridging the gap until catabolic processes such as glycogenolysis, glycolysis, and oxidative phosphorylation are engaged [9]. Arginine kinase is known to play essential roles in energy buffering, regulation of cell growth, and cellular defense against oxidative stress [10,11].

Accordingly, phosphagen kinases, including arginine kinase, play a critical role in maintaining energy homeostasis in tissues with high and fluctuating energy demands, such as neuronal tissue, gills, retinas, spermatozoa, and muscle fibers [1]. Various compounds—including arginine, creatine, lombricine, and glycocyamine—serve as phosphagen substrates for these enzymes [12]. While most phosphagen kinases function as single-domain enzymes, recent studies have identified a growing number of polydomain phosphagen kinases composed of multiple homologous domains [4]. This structural organization is believed to enhance enzymatic activity and efficiency through cooperative interactions between the tandem domains [13].

Arginine kinase and its closely related phosphagen kinase homologs play a pivotal role not only in short-term ATP regeneration but also in the spatial translocation of energy across different cellular compartments [14]. A wide range of endergonic cellular processes—such as molecular motion, active transport, biosynthetic metabolism, and signal transduction—are powered by nucleotide hydrolysis [15]. Therefore, the proper regulation of these enzymes is essential for maintaining cellular homeostasis [16].

Creatine kinase, primarily found in vertebrates, and arginine kinase, mainly present in invertebrates, contribute significantly to this regulation [2]. Arginine kinase does not exist in mammals, but it does exist in invertebrates that are human pathogens, so it could be used as a structure-based design template for new drug development [3]. These kinases function through reversible phosphate transfer reactions [17]. Creatine kinase is also closely associated with ATP-dependent processes and catalyzes the reversible transfer of a phosphate group between ATP and creatine [18]. It is known that lysine and arginine residues interact with the negatively charged phosphate groups of ATP [19]. While the role of a conserved cysteine residue has traditionally been implicated in stabilizing the nucleotide binding pocket rather than directly participating in catalysis. It may actually enhance catalytic turnover by electrostatically stabilizing the transition state, highlighting its importance in catalysis rather than in simple substrate binding [20,21].

Structural investigations have largely focused on vertebrate creatine kinases and invertebrate arginine kinases [4]. Although subunit architecture exhibits minor variations among different structures, they share a common structural framework consisting of a small N-terminal helical domain connected via a flexible linker to a larger C-terminal domain composed of β-sheets flanked by α-helices [5]. The substrate-free and transition-state analog structures show loop displacements of more than 10 Å and domain rotations of 17 degrees [22].

The observed structural diversity among homologs is primarily attributed to differences in oligomeric states and subcellular localization, such as cytosolic versus mitochondrial intermembrane compartmentalization [23]. Please note that this review paper focuses heavily on protein structural findings and only slightly reflects other biological data.

In this review, three key terms are frequently used for clarity: ‘transition-state analog’ refers to an enzyme–ligand complex that approximates the unstable, high-energy transition state formed during phosphoryl transfer. The terms ‘open conformation’ and ‘closed conformation’ describe the structural states of these kinases before and after ligand binding, respectively. In the open state, the active-site cleft remains accessible, while ligand binding induces a closed state through loop movement that shields the active site and aligns catalytic residues for phosphoryl transfer.

## 2. Results

To better understand the mechanistic and evolutionary aspects of phosphagen kinases, we examined high-resolution structures of arginine kinase and its homologs from a wide range of organisms, including vertebrates, invertebrates, and bacteria [4]. These enzymes catalyze the reversible transfer of a phosphate group between ATP and guanidino compounds such as arginine, creatine, lombricine, and glycocyamine [12]. While their catalytic roles are broadly conserved, individual arginine kinase s exhibit distinct structural adaptations depending on species, subcellular localization, and regulatory context [14]. This section presents a comparative overview of representative arginine kinase structures, including both substrate-free and transition-state analog complexes, to highlight key conformational changes during catalysis. We also explore unique structural features revealed through mutagenesis studies and electron microscopy, such as the activation mechanism of the bacterial kinase McsB by McsA. Together, these findings offer valuable insight into the structural basis of substrate binding, enzymatic regulation, and energy management across diverse biological systems.

Our approach aims to uncover unifying structural principles and mechanistic insights across diverse species. This section integrates recurring structural and mechanistic themes observed across the phosphagen kinase family. Rather than focusing solely on isolated examples, we emphasize shared features, distinct conformational transitions, and comparative discussions. By critically synthesizing structural data including apo forms, transition-state analogs, and ligand-bound variants, we aim to highlight novel patterns and regulatory mechanisms not previously integrated in the literature. These elements collectively offer a unified understanding of how structure underpins enzymatic function and evolutionary adaptation.

### 2.1. Bovine Retinal Brain-Type Creatine Kinase

During the initial phase of structural characterization of arginine kinase, the three-dimensional structure of cytosolic bovine retinal creatine kinase was successfully elucidated in 2001 [6]. Creatine kinase is a key enzyme that facilitates the reversible transfer of a phosphate group from phosphocreatine to ADP, leading to the formation of ATP and creatine [18].

**Figure 1:**
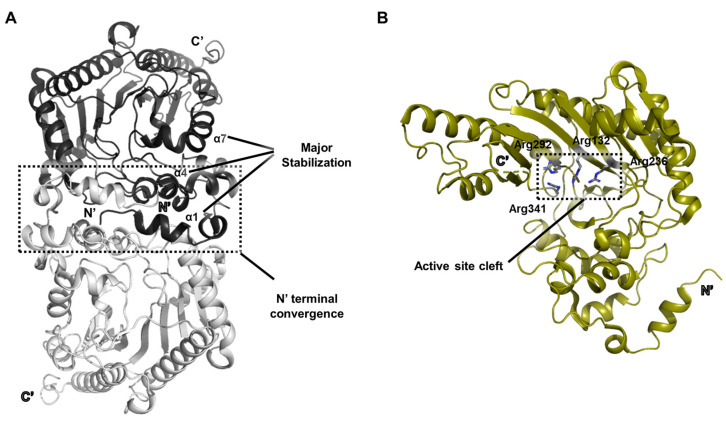
Overall description of the bovine retinal brain-type creatine kinase protein structure. (**A**) A structural view illustrating the overall symmetry of the dimeric enzyme. The dimerization interface originating from the N-terminal region is indicated, along with the major secondary structure elements contributing to its stabilization. These features are annotated for a single protomer only. (**B**) Architecture of the active site cleft enriched in multiple arginine residues within the same protein. As in (**A**), the structural annotations are shown for one monomeric unit.

The crystal structure of bovine creatine kinase was determined at a resolution of 2.3 Å (PDB ID: 1G0W) [4]. The biologically functional dimer adopts a symmetric configuration aligned along a crystallographic twofold axis (Figure 1A). Within the dimer interface, the N-terminal regions of each monomer converge near the symmetry axis, playing a stabilizing role in dimer formation. Intermolecular contacts involving α1 and α7 helices, together with interactions between a flexible loop and the α4 helix, constitute the main stabilizing forces for dimer integrity.

Structural analysis revealed a cluster of arginine residues located in a strongly electropositive region of the protein’s globular domain, defining the enzyme’s active site. This cleft, referred to as the active site cleft, comprises catalytically important residues such as Arg132, Arg236, Arg292, and Arg341 (Figure 1B).

The structural insights obtained from this dimeric arrangement provided a valuable framework for understanding the catalytic mechanism of creatine kinase, particularly regarding substrate positioning and phosphate transfer within the active site.

In bovine brain-type creatine kinase, the cluster of arginine residues within the active site cleft establishes a strongly positive electrostatic environment that plays a direct role in catalysis. These arginine side chains stabilize and orient the negatively charged phosphate groups of ATP and phosphocreatine, reducing the activation barrier for phosphoryl transfer. By anchoring the substrates through ionic and hydrogen-bonding interactions, they ensure precise positioning of the donor and acceptor groups, facilitating in-line transfer of the γ-phosphate. This local electrostatic environment contributes to stabilizing the transition state, thereby enhancing catalytic efficiency.

### 2.2. Rabbit Muscle Creatine Kinase

To investigate the conformational dynamics associated with catalytic function, the crystal structure of a transition-state analog complex of rabbit muscle creatine kinase was resolved at a high resolution of 1.65 Å (PDB ID: 1U6R) [24]. This structural model provides valuable insight into the molecular rearrangements required to facilitate and regulate the enzymatic reaction.

**Figure 2:**
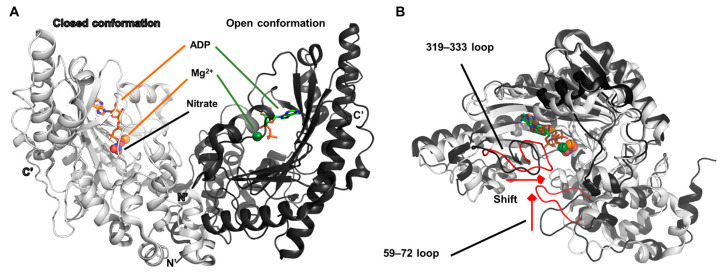
Structural features of rabbit muscle creatine kinase and ligand-dependent conformational shifts. (**A**) The asymmetric unit of the dimeric structure is shown. The monomer bound to nitrate is referred to as the “closed conformation,” while the monomer lacking nitrate is designated as the “open conformation.” In the closed state, ADP and Mg^2+^ are depicted in orange tones, whereas in the open state, the corresponding ligands are shown in green tones. (**B**) Structural alignment of the open (black) and closed (white) monomers. Two flexible loops responsible for driving the conformational transition are highlighted in red in the closed conformation. The viewing angle was adjusted to optimize the visualization of loop shift during the conformational shift.

In this study, the active site of one monomer was found in a closed conformation, stabilized by the simultaneous binding of magnesium ions, ADP, and nitrate—components that collectively mimic the transition state of the phosphotransfer reaction. In contrast, the opposing monomer within the dimer adopted an open conformation, where only Mg^2+^ and ADP were bound, and nitrate was absent (Figure 2A).

A comparative analysis between these two distinct conformations revealed that two flexible loop regions (residues 59–72 and 319–333) within the protein architecture play a critical role in modulating substrate access to the active site (Figure 2B). These loops undergo substantial conformational shifts, effectively functioning as a dynamic gate that either exposes or shields the catalytic cleft depending on the ligand occupancy. Such loop-mediated gating is proposed to be a key regulatory mechanism enabling precise control over the catalytic cycle of creatine kinase.

### 2.3. Trypanosoma cruzi Argininine Kinase

**Figure 3:**
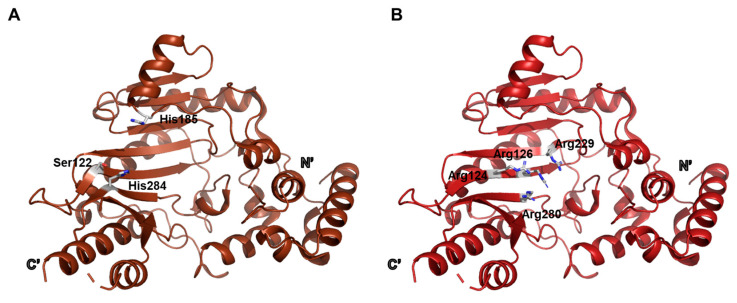
Structural features of the ligand-free open form. (**A**) Four residues predicted to participate in interactions with the nucleotide ring are highlighted. (**B**) Four positively charged residues forming a cluster that is expected to facilitate ATP access to the active site are shown.

The three-dimensional crystal structure of ligand-free *Trypanosoma cruzi* arginine kinase was determined at a high resolution of 1.9 Å, representing the open, unbound conformation of the enzyme (PDB ID: 2J1Q) [3]. This structure provides valuable insight into the inactive state prior to substrate binding. Importantly, several residues known to participate in nucleotide ring interactions—namely Ser122, His185, and His284—were observed to be conserved (Figure 3A). In addition, a positively charged cluster composed of Arg124, Arg126, Arg229, and Arg280, which is implicated in guiding ADP/ATP into the active site, was also well preserved (Figure 3B).

The flexible loop spanning residues 310–320, which plays a key role in modulating access to the active site, could not be clearly observed in this structure due to missing electron density. However, this loop is thought to undergo conformational changes upon ligand binding, acting as a dynamic gate that facilitates closure of the active site. Such a mechanism is presumed to be advantageous in preventing wasteful ATP hydrolysis under ligand-free conditions, thereby contributing to the regulation of catalytic efficiency [5].

### 2.4. The Structure of Lombricine Kinase from the Urechis caupo

Lombricine kinase, a member of the phosphagen kinase family, shares significant homology with arginine kinase and is introduced here for its role in regulating intracellular ATP levels. In this study, two structural forms of lombricine kinase from the marine annelid *Urechis caupo* were determined by X-ray crystallography (PDB ID: 3JPZ and 3JQ3) [25]. One structure represents a nucleotide-bound complex, while the other corresponds to the substrate-free state. Notably, the current analysis emphasizes the substrate-free conformation.

**Figure 4:**
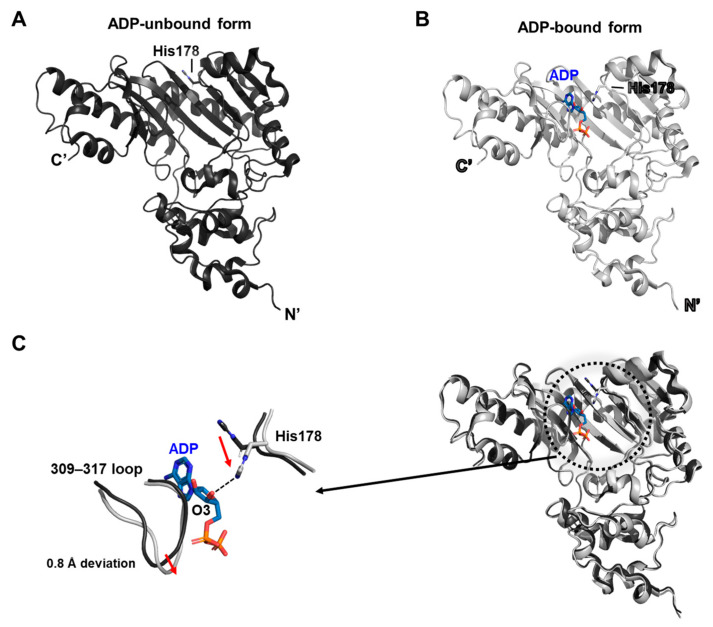
Summary of the kinase structure from *Urechis caupo*. (**A**) Structure of the ADP-unbound form. (**B**) Structure of the ADP-bound form, with His178 indicated as the key residue mediating ADP interaction. (**C**) Superposition of (**A**,**B**). The highlighted region, marked by a dotted circle, is enlarged to emphasize the hydrogen bond between ADP-O3 and His178. The conformational deviations of His178 and the dynamic loop upon ADP binding are illustrated with red arrows.

Interestingly, the ADP-bound structure of lombricine kinase maintains an open conformation, in contrast to the closed forms commonly observed in previously characterized kinases (Figure 4A,B). This unusual behavior is hypothesized to be associated with the specific interaction mode of His178 with the substrate. A loop previously described as dynamic in homologous enzymes exhibits a deviation of approximately 0.8 Å in this structure. Notably, the side chain of His178 is flipped toward the ribose ring of ADP, forming a hydrogen bond that appears to stabilize the ligand interaction. This conformational change likely propagates through the enzyme and contributes to shaping the overall nucleotide-binding site (Figure 4C).

The 309–317 loop, a known substrate-specificity region, has been characterized by NMR as intrinsically flexible. The authors propose that His178 functions not merely as a passive structural mediator, but rather as a stochastic modulator that reflects a dynamic equilibrium among multiple conformational states, rather than enforcing a singular structural transition.

### 2.5. Structural Basis of Glycocyamine Kinase from the Marine Worm Namalycastis sp

**Figure 5:**
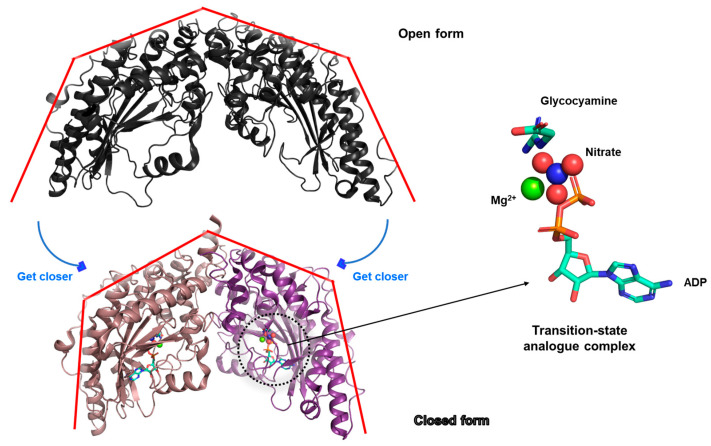
Observation of conformational changes induced by binding of the transition state analog complex. The ligand-free form is shown in black cartoon (upper), while the ligand-bound form is depicted in color cartoon (lower). To aid in visualizing the conformational shift toward the closed form upon ligand binding, several red broken lines and blue directional arrows are included. The individual components of the transition state analog complex are additionally labeled in detail on the right. Note that although both monomers are bound to the substrate, only one monomer is shown and enlarged for clarity in the drawing.

By the early 2010s, structural insights into glycocyamine kinase—an enzyme belonging to the phosphagen kinase family that catalyzes Mg^2+^-dependent ATP-generating reactions—had been successfully obtained (PDB ID: 3L2F) [26] using X-ray crystallography. This kinase facilitates the transfer of a phosphoryl group from phosphoglycocyamine to ADP, playing a critical role in cellular energy homeostasis. A key breakthrough in structural understanding involved the characterization of the enzyme in complex with a four-part molecular assembly: Mg^2+^, ADP, nitrate and glycocyamine. This specific combination is referred to as a transition state analog, designed to mimic the high-energy transition state encountered during phosphoryl transfer (Figure 5).

When this transition state analog binds to the enzyme, a significant conformational shift is triggered—one that effectively brings the two structural lobes of the enzyme closer together. The protein appears to close inward around the bound transition state analog. As a result, researchers classify this ligand-bound form as the “closed” conformation. In contrast, the unbound or ligand-free enzyme adopts an “open” conformation, where enzymes are more spatially separated. This open–closed dynamic provides critical insight into the enzyme’s mechanism and its regulation by substrate binding (Figure 5).

### 2.6. Arginine Kinase from Anthopleura japonicus

The authors of this study propose that the monomeric structure is composed of two distinct domains, and they interpret the interdomain cooperativity observed in such polydomain enzymes as a structural basis for synergistic catalytic mechanisms [27]. From an evolutionary standpoint, if a polydomain enzyme merely functioned as a sum of its individual domain activities, it would provide no selective advantage and likely be eliminated through natural selection [28]. Therefore, polydomain enzymes such as the arginine kinase from *A. japonicus* are likely preserved due to the evolutionary benefit conferred by interdomain synergism [27].

**Figure 6:**
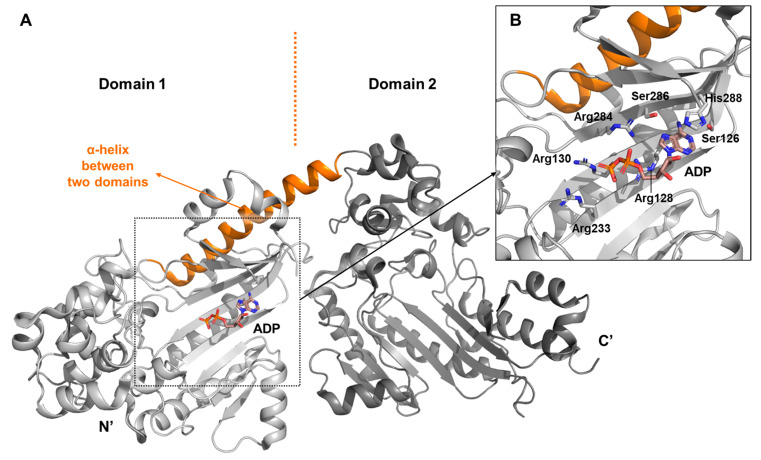
Summary of the arginine kinase structure from *A. japonicus*. (**A**) Unlike previously described kinases, this structure features two kinase domains arranged in a polydomain configuration within a single monomer. Each domain is shaded in varying intensities of gray to distinguish them. The α-helix that bridges the two domains, and the bound ADP, is highlighted in orange. Analysis of flexible loops has been omitted due to the lack of interpretable electron density in those regions. (**B**) Spatial arrangement of residues involved in the active site interaction with ADP. Since interactions with ADP are comparable to those observed with ATPγS, only the ADP-bound state is shown. Note that Arg233 does not directly contact ADP due to the spatial separation.

This particular arginine kinase comprises two kinase domains of approximately 40 kDa each: the N-terminal domain (D1, residues 1–368) and the C-terminal domain (D2, residues 369–715) (PDB ID: 4RF8) [27] (crystal structure) (Figure 6A). Unlike dimeric enzymes described earlier that exhibit pseudosymmetry, the two domains of this enzyme are connected in a notably asymmetric configuration. Unexpectedly, the interdomain connection is not formed by a flexible hinge region, but rather by a long, rigid α-helix that serves as a structural element to hold the two domains in relatively fixed positions.

At the D1 active site, ADP is coordinated by a cluster of positively charged residues, including Arg128, Arg130, Arg233, and Arg284, which engage the phosphate groups, while Ser126, Ser286, and His288 contribute to nucleotide stabilization through hydrogen bonding and π-π stacking interaction (Figure 6B). The nature of this interaction was further validated by binding studies using ATPγS, a non-hydrolyzable analog in which the terminal phosphate oxygen is replaced with sulfur, confirming the identity and positioning of the key binding residues.

### 2.7. Arginine Kinase from the Horseshoe Crab Limulus polyphemus

**Figure 7:**
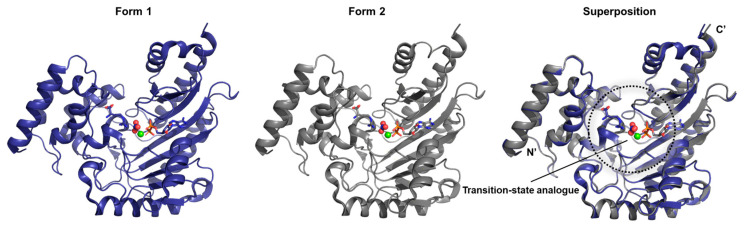
Shown are the two structural forms of *L. polyphemus* arginine kinase, each complexed with a transition state analog. Both forms were determined independently but exhibit highly similar conformations. As discussed in the main text, structural differences between the two are minimal.

In this study, two structural variants of the transition state analog complex of arginine kinase derived from *Limulus polyphemus*—designated as Form I and Form II—were resolved (PDB ID: 5J99 and 5J9A) [22] by crystallography (Figure 7). According to the authors, efforts to crystallize this enzyme spanned several years, and among the crystals obtained, the ratio of Form I to Form II was approximately 1:3. Despite arising under the same experimental conditions, the structural distinctions between these two forms were minimal. Subdomain rotations, as defined by the authors’ internal domain classification, were reported to range only between 0.4° and 3.5°, suggesting subtle bending differences. Furthermore, the root mean square deviation (RMSD) between the backbone and Cβ atoms of the two forms was calculated to be 0.29 Å, a value only marginally higher than the empirically estimated crystallographic error of 0.24 Å. Taken together, these observations indicate that Form I and Form II are structurally nearly identical.

### 2.8. Crystal Structure of Scylla paramamosain Arginine Kinase

Crustacean-derived arginine kinase is recognized as a potent allergen with strong IgE reactivity [29]. This allergenic property has also been identified in arginine kinases from other invertebrates such as mites, cockroaches, spiders, and octopuses [30,31,32]. In the present study, the three-dimensional structure of arginine kinase from *S. paramamosain* was determined by X-ray crystallography following protein purification (PDB ID: 5ZHQ) [33]. Notably, instead of employing a recombinant expression system, the protein was directly extracted from crab muscle tissue and subsequently processed to obtain crystallizable samples. The use of naturally sourced arginine kinase adds a unique feature.

**Figure 8:**
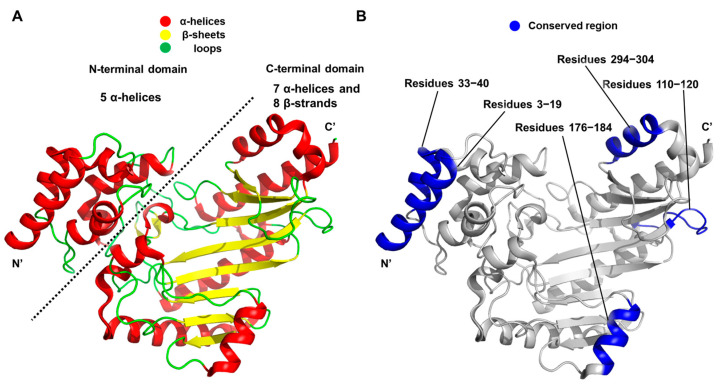
Structural analysis of arginine kinase from the crab. (**A**) Secondary structure elements are illustrated with distinct colors for each structural motif. The N-terminal and C-terminal domains discussed in the main text are also visualized. (**B**) Conserved regions are displayed as shaded areas in blue, accompanied by their corresponding residue number ranges.

The *S. paramamosain* arginine kinase exhibits the characteristic fold observed in other arginine kinases, comprising an N-terminal domain formed by five α-helices and a C-terminal domain with a mixed arrangement of seven α-helices and eight β-strands (Figure 8A). As of 2019, accumulated structural data have revealed that certain regions—namely residues 3–19, 33–40, 110–120 (loop region), 176–184 and 294–304—are well conserved across arginine kinases in terms of both primary sequence and three-dimensional structure (Figure 8B). It is statistically shown to exhibit high sequence and structural conservation among various arginine kinases.

### 2.9. Insight into Structural Aspects of Daphnia magna Arginine Kinase

In the 2020s, structural studies incorporating mutational analyses—previously unexplored—have begun to emerge [34]. In this study, the role of His284, a highly conserved residue in *D. magna* arginine kinase, was investigated through site-directed mutagenesis. Until that point, the function of His284 had not been elucidated.

**Figure 9:**
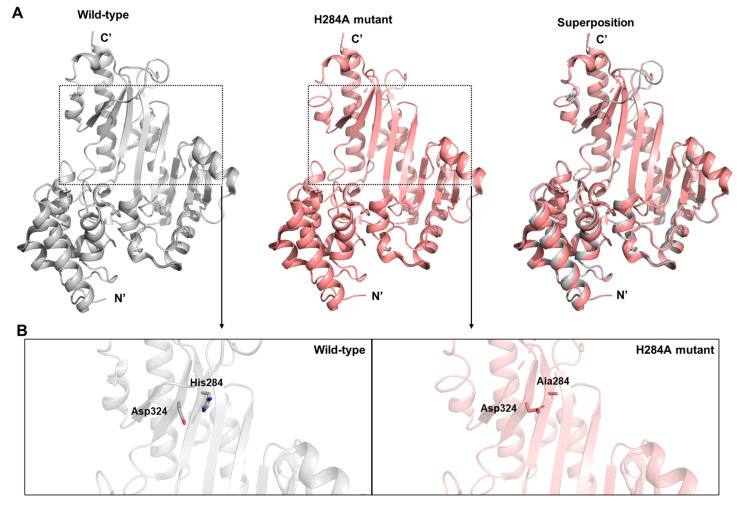
Structural characterization of *D. magna* arginine kinase. (**A**) From left to right: crystal structures of the wild type (WT), H284A mutant, and their superposition. As shown, the overall structural difference between the two is minimal, consistent with the r.m.s.d. values reported in the main text. (**B**) Comparison of Asp324 positions in the WT (left, gray) and H284A mutant (right, pink). The authors propose that this shift alters the local environment surrounding His284 or Ala284. Note the distinct orientations of Asp324 in the two structures.

The overall crystal structures of wild-type (WT) and the H284A mutant showed no substantial deviation, with an r.m.s.d. of 0.22 Å based on Cα atoms (PDB ID: 6KY2) [34] (Figure 9A). However, the H284A mutation led to several localized structural changes, including a rotameric shift in Asp324 and reorganization of the interaction network surrounding the mutated site. Comparison with the WT structure further revealed that, in the H284 mutant, the carboxyl group of Asp324 adopted a new orientation toward Ala284, suggesting a rotameric change with potential consequences for the local hydrogen-bonding environment (Figure 9B).

### 2.10. Structure of Arginine Kinase McsB from Staphylococcus aureus

In recent years, the structural elucidation of bacterial protein arginine kinases has begun to emerge. One such enzyme, McsB, plays a crucial role in protein repair under stress conditions by acting as a degradation labeler—tagging damaged or misfolded proteins for clearance [35]. McsB is a bacterial kinase with a unique enzymatic capability to phosphorylate arginine residues on protein substrates, forming a central component of the bacterial protein quality control system [36]. Upon activation by its cofactor McsA, McsB phosphorylates arginine residues on transcriptional repressors, thereby inactivating them and triggering the expression of genes essential for kinase activity and stress adaptation [35,36].

**Figure 10:**
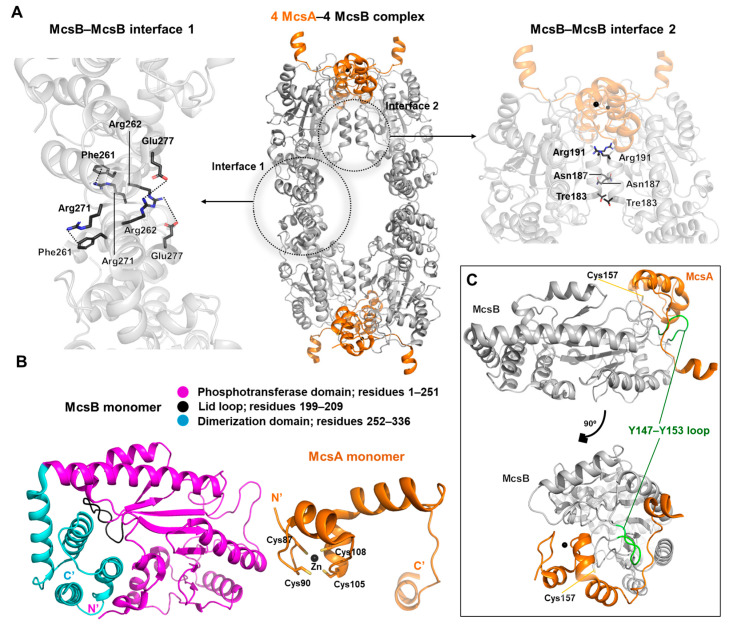
Structural details of the McsA–McsB complex and individual McsA and McsB components. (**A**) (center) The McsA–McsB complex is shown, with McsA colored in orange and McsB in gray. Four McsA and four McsB subunits form a tetrameric assembly. As indicated by the dotted circles, two distinct types of McsB–McsB homodimeric interfaces are present. The salt bridges, hydrogen bonds, and hydrophobic networks involved in each interface are illustrated in detail on the left and right panels. (**B**) Isolated structures of McsB and McsA. (left) McsB is shown from the N-terminus with the P domain in purple, lid loop in black, and D domain in cyan. (right) McsA is shown with its four conserved cysteine residues and a bound zinc ion. (**C**) Heterodimeric McsA–McsB interaction. Two rotated views are presented for optimal visualization. The McsA zinc finger (orange), McsB catalytic residue Cys157 (yellow), and the extended loop (green) are highlighted.

This structure represents the only electron microscopy-derived architecture described in this review (PDB ID: 8GQD) [35]. Specifically, it captures the tetrameric assembly of the McsA–McsB complex (Figure 10A). Tetramer formation is reported to be essential for McsB’s catalytic function [35]. Moreover, McsA binding enhances the thermal stability of McsB, contributing to the maintenance or enhancement of its enzymatic activity. The interface between heterodimers within the tetramer is stabilized primarily by the salt bridge formed between Arg262 and Glu277, and by a combination of aromatic stacking and cation–π interaction between Phe261 and Arg271.

Structurally, McsB is composed of a dimerization domain (DD; residues 252–336), a phosphotransferase domain (PD; residues 1–251), and a flexible ’lid’ loop (residues 199–209). McsA, on the other hand, harbors four conserved cysteine residues (Cys87, Cys90, Cys105, and Cys108) within a zinc finger domain that coordinates a zinc ion (Figure 10B).

The mechanism by which McsA activates McsB involves interaction between the C-terminal zinc finger of McsA and an extended loop region (Y147–Y153) of McsB. This interaction serves to properly orient the catalytically essential cysteine residue, Cys157, optimizing its spatial positioning for enzymatic function (Figure 10C).

### 2.11. Comparative Structural Themes Across Phosphagen Kinases

This review organizes the enzymes of the phosphagen kinase family according to the chronological order in which their structures were elucidated. However, such an approach can easily appear as a descriptive catalog of individual structures, and therefore an integrative discussion of structural themes across the family is essential. By comparing the structures described, we aimed to provide insights that extend beyond previously reported observations.

Across the many species examined, phosphagen kinases share a broadly conserved overall architecture and exhibit similar patterns of structural change during catalysis. Ligand binding consistently drives a shift from an open to a more compact form, bringing the two major parts of the enzyme closer together. This motion is accompanied by the rearrangement of flexible surface regions that act like gates to control access to the active site. Despite differences in sequence and origin, these enzymes maintain a common structural strategy for energy transfer, illustrating the close relationship between their form, motion, and catalytic function.

**Figure 11:**
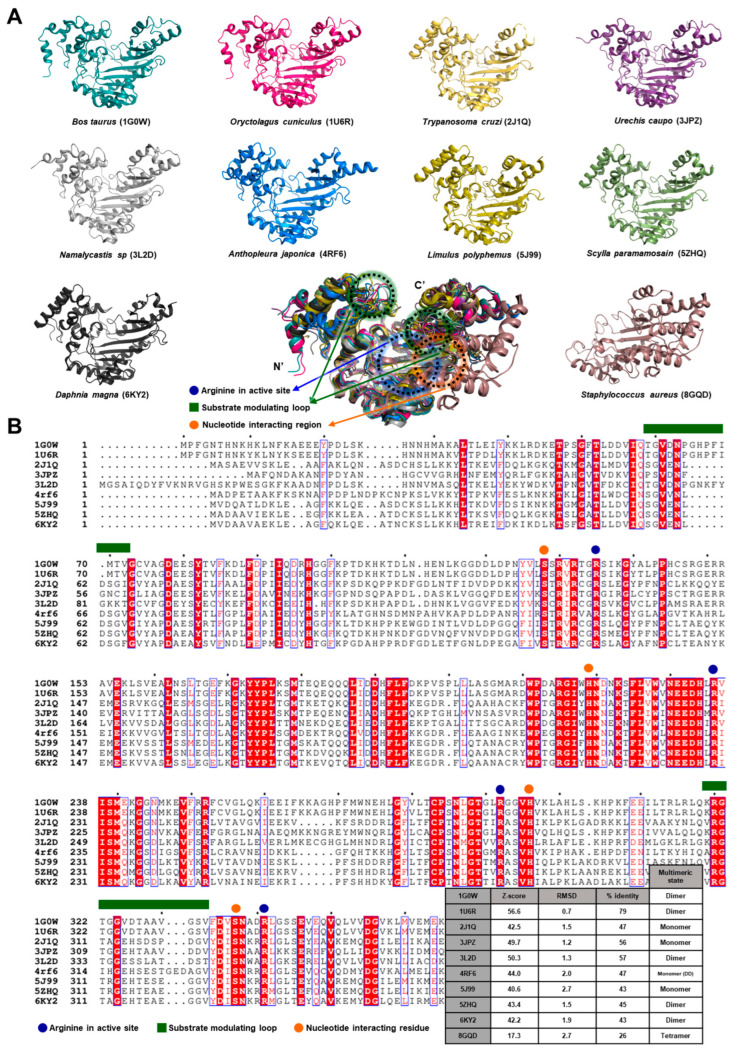
Comparative structural themes across phosphagen kinases. (**A**) Structural comparison among kinases. Structural themes described in Section 2.1, Section 2.2, Section 2.3, Section 2.4, Section 2.5, Section 2.6, Section 2.7, Section 2.8, Section 2.9 and Section 2.10 are shown in distinct colors. In the overlay diagram at the bottom, the approximate locations of the active site enriched in arginine residues (blue), the flexible loop that modulates substrate binding (green), and the region primarily interacting with nucleotides (orange) are indicated with dotted circles. (**B**) Sequence comparison among kinases. Oligomeric states and key structural parameters are indicated at the lower right. Note that 8GQD, which is structurally less similar to the others, was excluded from the sequence alignment for clarity. As in panel (**A**), the same color coding is used to annotate corresponding residues or regions within the comparison diagram.

The analyzed structures share a broadly similar overall fold and exhibit recurring architectural features, including an active site enriched with arginine residues, flexible loops that regulate substrate binding, and regions that primarily interact with nucleotide ligands (Figure 11A). These common elements become even more evident when viewed through sequence alignments, highlighting the evolutionary conservation of these enzymes across organisms that are otherwise distantly related (Figure 11B). This conservation underscores their fundamental role in cellular energy regulation and related physiological processes.

**Figure 12:**
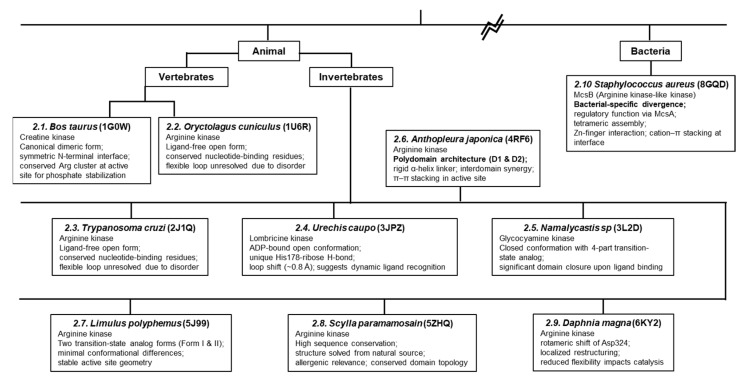
Phylogenetic tree of representative phosphagen kinases analyzed in this review. Note that this tree has been simplified according to minimal evolutionary relationships to efficiently reflect the divergence of organisms described in this review. Vertebrate creatine kinases form a distinct clade, while invertebrate arginine and related kinases display various structural adaptations. Structural features such as domain architecture, catalytic loop mobility, and ligand-binding mode are annotated for each kinase, illustrating evolutionary pressures shaping functional specialization. Key features described in the main text are indicated in bold.

Across the phosphagen kinase family, structural conservation is achieved by lineage-specific adaptations that reflect distinct physiological and evolutionary pressures. For example, while the core catalytic fold remains stable, variations in domain organization such as the emergence of polydomain forms in *A. japonicus* suggest evolutionary selection for synergistic catalysis. Similarly, bacterial homologs like McsB have diverged significantly to acquire regulatory functions distinct from canonical energy buffering. To visualize these relationships, we constructed a phylogenetic tree annotated with key structural features (Figure 12). This tree illustrates how evolutionary branching correlates with shifts in domain complexity, catalytic loop architecture, and ligand interactions, highlighting the adaptive plasticity of this enzyme family.

## 3. Conclusions

This review has explored the structural and mechanistic diversity of arginine kinases and related phosphagen kinases across a broad phylogenetic spectrum. Despite their conserved catalytic function—phosphoryl transfer between ATP and guanidino substrates—these enzymes exhibit remarkable structural variability that reflects evolutionary and functional specialization.

A key insight across multiple species is the shared bipartite structure of arginine kinases, composed of a smaller N-terminal domain and a larger C-terminal phosphotransferase domain. The ligand-bound and ligand-free structures highlight the importance of flexible loop regions and domain movements in controlling access to the active site. Mutagenesis studies, such as those involving His284 in *Daphnia magna*, further demonstrate how subtle changes near the catalytic core can significantly impact enzymatic efficiency by altering local interactions and flexibility.

Bacterial arginine kinase-like enzymes, especially McsB, deviate structurally from canonical phosphagen kinases but fulfill analogous functions in protein quality control. The activation of McsB by McsA through zinc finger-mediated structural stabilization exemplifies a novel regulatory strategy. Notably, McsA not only facilitates the correct positioning of catalytic residues but also prevents undesired oligomerization, underscoring its essential role in modulating McsB activity.

Arginine kinase has gained increasing attention in the context of disease, particularly due to its role as a potent allergen and immunogenic protein in invertebrates. Crustacean and insect arginine kinases are recognized as major IgE-binding allergens, contributing to respiratory and food allergies in sensitized individuals. Structural studies have revealed conserved epitopes that underlie the strong cross-reactivity between species such as shrimp, crabs, spiders, and mites. These insights have important implications for allergic disease, providing a molecular basis for diagnostic epitope mapping and suggesting structure-guided approaches for desensitization or immunotherapy. Thus, beyond its metabolic function, arginine kinase is directly linked to clinically relevant hypersensitivity disorders [37,38,39].

Overall, the structural insights detailed in this review deepen our understanding of how phosphagen kinases accommodate functional demands in diverse organisms. These findings are not only valuable for basic enzymology and comparative biochemistry but also provide a solid framework for applied research, including allergen prediction, drug design, and vector-borne disease diagnostics. As structural data continue to accumulate, the full spectrum of regulatory adaptations among these enzymes is expected to become even clearer.

## Data Availability

No new data were created or analyzed in this study. Data sharing is not applicable to this article.

## Abbreviations

The following abbreviations are used in this manuscript:

ATP	Adenosine Triphosphate
RMSD	root mean square deviation
WT	wild type
